# Neurotransmitters of the suprachiasmatic nuclei

**DOI:** 10.1186/1740-3391-4-2

**Published:** 2006-02-16

**Authors:** Vallath Reghunandanan, Rajalaxmy Reghunandanan

**Affiliations:** 1 Department of Basic Medical Science, Faculty of Medicine and Health Sciences, University of Malaysia, 93150 Kuching, Malaysia

## Abstract

There has been extensive research in the recent past looking into the molecular basis and mechanisms of the biological clock, situated in the suprachiasmatic nuclei (SCN) of the anterior hypothalamus. Neurotransmitters are a very important component of SCN function. Thorough knowledge of neurotransmitters is not only essential for the understanding of the clock but also for the successful manipulation of the clock with experimental chemicals and therapeutical drugs. This article reviews the current knowledge about neurotransmitters in the SCN, including neurotransmitters that have been identified only recently. An attempt was made to describe the neurotransmitters and hormonal/diffusible signals of the SCN efference, which are necessary for the master clock to exert its overt function. The expression of robust circadian rhythms depends on the integrity of the biological clock and on the integration of thousands of individual cellular clocks found in the clock. Neurotransmitters are required at all levels, at the input, in the clock itself, and in its efferent output for the normal function of the clock. The relationship between neurotransmitter function and gene expression is also discussed because clock gene transcription forms the molecular basis of the clock and its working.

## Introduction

Great advances have been made in the study of mechanisms of the circadian clock in the past decade. Since the identification of a master circadian clock in the suprachiasmatic nuclei (SCN) of the anterior hypothalamus of mammals, researchers sought to identify the nature of the clock and characterize its components. The SCN, acting as circadian pacemakers, have the function of orchestrating the timing in physiology and behaviour. They control circadian rhythms in other parts of the brain, such as the cerebral cortex, in the pineal gland, and in peripheral tissues such as liver, kidney and heart [1]. The circadian clock not only can generate its own rhythms but can also be entrained by the environmental light-dark (LD) cycle. Multiple single cell circadian oscillators that are present in the clock can, when synchronized, generate coordinated circadian outputs which ultimately regulate the overt rhythms.

Studies pertaining to the molecular mechanisms of the clock have yielded valuable results with the identification of a protein responsible for the setting of the length of periods of activity and inactivity within cells. Many years of research by a dedicated team of scientists culminated in the discovery of this protein [2]. It is believed that the identification of this protein will have far reaching implications not only in the understanding of the working of the clock but also in clinical applications, such as the treatment of jet lag and the design of optimal times for the administration of anti-cancer drugs.

The master clock, as it is often called, is reset by light or photic stimuli [3] as well as by arousal-inducing or non-photic stimuli [4]. Whether the input is photic or non-photic, it reaches the clock through neurotransmitters in nerve terminals. Neurotransmitters are released at the inputs for entrainment, in the clock itself for integration and consolidated output, and in efferent projections for the control of overt rhythms. Several reviews on the neurotransmitters of the SCN have been previously published [5-10]. The present review concentrates on studies conducted in the last decade and gives particular attention to neurotransmitters whose involvement in the circadian clock have not been traditionally recognized.

### About neurotransmitters in general

Studies have indicated the presence of a large number of neurotransmitters in the SCN [11-15]. However, information about their role individually as well as in combination in the functioning of the clock has been slow to come. It is observed that the presence of neurotransmitters in the afferent and efferent projections of the SCN is equally important for the entrainment of the clock and for the control of overt rhythms. Thus, we have neurotransmitters released at the inputs for entrainment, in the clock itself for the integration and consolidated output, and in the efferent projections for the control of overt rhythms.

There have been attempts to categorize the putative neurotransmitters of the SCN on the basis of their origin and function [16] and there have been reports indicating subdivisions of the SCN with relation to neurotransmitter function [17]. Further, it has been reported [18] that the human SCN also have well defined subdivisions with chemically defined neuronal groups comparable to the well defined subdivisions reported in the case of experimental animals, mainly rodents. There are many excellent reviews [19-22] highlighting various aspects of the neurotransmitters. From a functional point of view, two important aspects emerge. One is the fact that one particular neurotransmitter may have more than one function and thereby make the prediction of the function more difficult and complex. Another aspect is that the neurotransmitter input from various pathways and their influence may vary (1) by itself and (2) by way of modification of SCN function. Neurotransmitters like acetylcholine, glutamate, neuropeptide Y (NPY), serotonin, vasoactive intestinal peptide (VIP), peptide histidine isoleucine (PHI), and arginine vasopressin (AVP) have been implicated in the functioning of the SCN. Glutamate and pituitary adenylate cyclase-activating polypeptide (PACAP) are indicated as principal neurotransmitters of the retinohypothalamic tract (RHT), although excitatory amino acids like L-aspartate and N-acetyl-aspartylglutamate may also function as neurotransmitters in RHT. Substance P also might be a candidate as a neurotransmitter in RHT. Functional studies over the years have given evidence that PACAP alone or in concert with glutamate may be responsible for the light signalling to the clock.

The role of AVP in circadian time keeping has been well established. Its role in the control of circadian rhythm of food and water intake has been reported and well documented. Another intrinsic neuropeptide, VIP, acting through VPAC_2 _receptor (a type of receptor for VIP), participates in both resetting to light and maintenance of ongoing rhythmicity of the SCN. NPY and GABA seem to be the neurotransmitters in the projection from the intergeniculate leaflet to the SCN. Raphe nuclei projections to the SCN contain serotonin. AVP and prokineticin 2 are seen in the outputs from the SCN.

The neurotransmitter-dependent molecular basis of the working of the clock is yet to be understood completely. The specific roles of the various neurotransmitters may be based on the response of the neurons of the SCN on application of a neurotransmitter, capacity to phase shift a particular rhythm on application of neurotransmitter, effect of lesions on the entrained and free running rhythms, and disruptions seen in the rhythms after blockade by the antagonist/inhibitors. Possible targets for some of the neurotransmitters are the clock genes *per1 *and *per2*, which are induced in the SCN by light or by neurotransmitters, at night.

Evidence indicating co-localization of some of the neurotransmitters in the SCN has further complicated the investigations into the role of neurotransmitters in the working of the clock. It is likely that the functioning of the clock may depend on the presence of a particular neurotransmitter on a mechanism in which co-localized neurotransmitters interact in a functionally significant manner.

With more information available on the role of neurotransmitters in the working of the clock, which is involved in so many functions of the body, better opportunity for neurotransmitter-based manipulation of the clock has also been reported. Problems of shift-work insomnia and ill effects of jet lag are among the clock-related functions for which much attention has been given in recent years. Melatonin has been in use with some success in reducing the above effects. However, search for other chronobiotic agents is continuing and it is likely that there may be some new dimensions given to the problem and its solution in future. A report of a close relationship between circadian clock and cell proliferation makes things even more interesting. Investigations into the role of neurotransmitters in the SCN as well as in the afferent and efferent inputs have come a long way with the advent of newer techniques of positron emission transaxial tomography (PET) scan and polymerase chain reaction (PCR).

In both plants and animals, circannual rhythms are widely distributed. Endogenous circannual rhythms form the basis for many seasonal rhythms. A number of neuroendocrine mechanisms have been implicated in the regulation of seasonal changes in the physiology and behaviour of animals. Some of these neuroendocrine pathways are necessary for the regulation of particular overt seasonal responses, though they may not be directly linked to the circadian time-keeping system. Photoperiodic input and circannual function may have profound influence on many of the functions of body. Naturally, neurotransmitters are involved not only in circadian function but also in seasonal processes. It has been postulated that heterogeneity of the clocks which are seen within the SCN may be one of the factors that form the basis of seasonal adaptations [23]. A typical example of the close linkage between seasonal rhythms and affective disorders can be seen in a seasonal form of mood disorder, seasonal affective disorder (SAD). Treatment for SAD based on circadian principles includes not only light therapy but also the use of certain drugs, again based on circadian principles involving neurotransmitters.

The SCN has been subdivided into a dorsomedial shell and a ventrolateral core. This is based on retinal innervation patterns as well as the observation that these regions are defined by phenotypically distinct cell types [24]. Each nucleus contains about 10,000 neurons, thereby making 20,000 neurons in total. These neurons are characterized by small size and high density [25]. Isolated individual neurons are reported to produce circadian oscillations with periods ranging from 20–28 h [26,27]. Circadian oscillations are generated in the individual neurons of the SCN by a molecular regulatory network. Though individual cells oscillate with periods ranging from 20–28 h, at the tissue level SCN neurons display synchrony indicative of a robust inter-cellular coupling, and neurotransmitters appear to have an important role in the inter-cellular coupling. Gondze and co-workers [28] have introduced a molecular model for the regulatory network underlying the circadian oscillators in the SCN and stated that effective synchronization is achieved when the average neurotransmitter concentration damps the individual oscillators. Cells are effectively synchronized due to global neurotransmitter oscillation. Neither spiking in the neurons of the SCN nor chemically mediated transmission is needed for the pacemaking activity seen in individual cells. However, synchronization of circadian rhythmicity across neurons in the SCN does require neurotransmitters [25,29] and development of action potentials [30].

Before attempting a discussion of the neurotransmitters, it is necessary to identify the afferent projections to the SCN, and neurotransmitters present in it, neurotransmitters intrinsic to the SCN, and efferent projection with its neurotransmitters. The SCN is composed of different neuronal elements, each having its own specific function. Intensive interconnection and interaction among the heterogeneous neuronal elements is responsible for the functional output of the SCN. Different neurons of the SCN contain different neuropeptides, with several neurons having co-localization of neurotransmitters. Thus, we have, for example, gamma amino butyric acid (GABA) and glutamate, GABA and AVP, AVP and corticotrophin releasing hormone (CRH), AVP and its carrier protein neurophysin, VIP and peptide histidine isoleucine (PHI), and VIP and somatostatin (SS). Co-localization of different neuropeptides is seen not only in rat SCN but also in human SCN [31-33]. The combination of a variety of peptides with or without amino acid neurotransmitters within a single nucleus gives the SCN a variety of signalling properties as well. A set of SCN neurons and their neurotransmitters has the function of conveying the daily light-dark signal to hypothalamic target structures [34-36].

**Figure 1:**
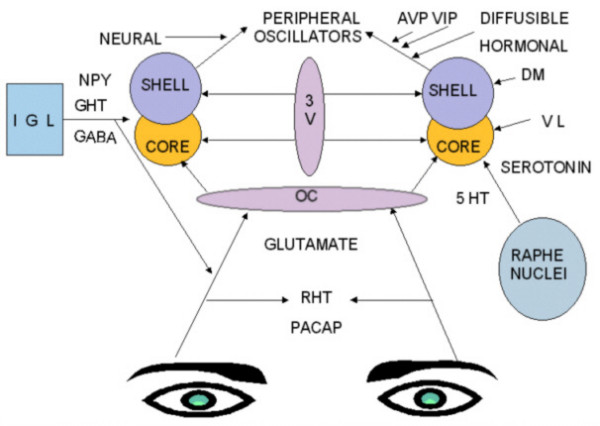
**Afferent inputs and efferent pathways of the SCN. **RHT: Retinohypothalamic tract, GHT: Geniculohypothalamic tract, OC: Optic chiasm, 3V: Third ventricle, IGL: Intergeniculate leaflet, DM: Dorsomedial SCN, VL: Ventrolateral SCN, NPY: Neuropeptide Y, GABA: Gamma amino butyric acid, PACAP: Pituitary adenylate cyclase-activating polypeptide.

Three major incoming pathways have been identified for the SCN. These have been defined as the retinohypothalamic tract (RHT), geniculohypothalamic tract (GHT), and the projection from the raphe nuclei (Figure 1). Photic information is relayed directly from the retina to the SCN by way of the monosynaptic retinohypothalamic tract [37,38]. It is seen that transection of all visual pathways leaving the optic chiasm makes animals blind with no visual reflexes but with perfect normal entrainment of circadian rhythms. This has indicated that RHT is sufficient for entrainment. Also it has been demonstrated that sectioning of RHT abolishes entrainment without affecting visual functions [39]. Although the exact mechanism by which a monophasic stimulus, light, produces either no response or a biphasic response in SCN neurons is unclear at present, Myers and co-workers [40] provided a potential molecular explanation for the phenomenon. Using electron microscopy and immunohistochemistry, they identified the excitatory amino acid glutamate and pituitary adenylate cyclase-activating polypeptide (PACAP) as the main neurotransmitters of the RHT [41-43]. Although substance P (SP) was thought of as a neurotransmitter of the RHT, there is now substantial evidence against this possibility [44-46].

## Retinohypothalamic tract (RHT) and its neurotransmitters

The principal neurotransmitters involved in conveying photic information to the SCN have been identified as glutamate and PACAP. Light stimulation of the retina results in direct secretion of glutamate from the RHT into the ventral VIP-containing part of the SCN [47-49]. Glutamate as a transmitter at RHT/SCN synaptic connections plays an important and critical role in mediating photic regulation of circadian rhythmicity. RHT terminals innervating the SCN show glutamate immunoreactivity associated with synaptic vesicles [41,50], which confirms the role of glutamate as a neurotransmitter. Different types of glutamate receptors were identified and localized in the SCN using in situ hybridization and immunocytochemistry [51]. PACAP, which is co-localized in a subpopulation of glutamate-containing retinal ganglion cells and also involved in relaying light information, may potentiate the action of glutamate on the SCN [52,53]. Both glutamate and PACAP fulfil the criteria of being located in the RHT, being released on stimulation, affecting the cells of the SCN in a manner similar to light, and having their effects blocked by specific antagonists [20]. Exogenous application of glutamate receptor (GluR) agonists is found to excite SCN neurons [54,55] and cause phase shifts. On the other hand, GluR antagonists block light-induced phase shifts and Fos-induction in the SCN in vivo [56,57].

### Nitric oxide (NO)

NO appears to be a crucial neuroactive substance for the function of the SCN. Presence of neurons showing nitric oxide synthase (nNOS) immunoreactivity in the SCN of dwarf hamster and rat [58,59] were further confirmed by the studies of Chen and co-workers [60] and Caillol and co-workers [61]. Nitric oxide production in the SCN has been linked to N-methyl D-aspartate (NMDA)-induced cyclic guanosine monophospahate (cGMP) production, and administration of cGMP produces phase shifts of circadian rhythms in vitro [62]. It has also been reported that NOS inhibitors prevent NMDA-induced phase shifts of circadian rhythms in vitro and in vivo [48]. There is also a possibility for an additional source of NO in the SCN from the astrocytes, as a group of cells positive for endothelial NOS (eNOS) was found in rat and hamster [61].

In terms of the functional impact of NO in the working of the SCN, blocking of NO production disrupts light transmission to the SCN [63], thus indicating the possibility of the role of NO in the light-input pathway. NO synthesis is required for phase changes of electrical activity [64]. Intracerebroventricular application of L-NAME (a drug that blocks NOS in hamsters) produces attenuation of light-induced phase-advances of activity rhythms [65]. Reports also indicate interruption in the light-triggered cascade of glutamate release from retinal terminals in the SCN by blockade of NO action in intact animals, which leads to subsequent interruption of NMDA receptor activation [66,67]. Interruption of intracellular increase of calcium, activation of nNOS, augmented production of cyclic guanosine monophosphate (cGMP), activation of protein kinase C, and phosphorylation of cyclic adenosine monophosphate (cAMP) response element binding protein (CREB), as well as interruption of the expression of immediate early genes, are other effects of blockade of NO action.

Starkey and co-workers [68] provided evidence for the presence of functional type II NOS within the SCN of guinea pig. All isotypes of NO synthase have also been identified in the normal adult mammalian SCN. Contribution by more than one NOS isotype to the regulation of circadian rhythms cannot be ruled out. In this context, it is interesting to note the demonstration by Kriegfeld and co-workers [69] that mice lacking the gene for type I NOS experience no change in the ability to phase-shift or entrain circadian rhythm of locomotor activity. Yet another study, also by Kreigfeld and co-workers [70], has suggested that endothelial isoform of NOS among the three known isoforms may not be necessary for photic entrainment in mice. However, considering the three different forms of NOS identified, until the isoforms of NOS involved in regulating the clock phase by modulating inputs are completely established it is difficult to speculate the exact role of NO.

There has been much speculation as to the photopigment mediating light information to the SCN. However, this is not yet known with certainty [71]. A novel opsin, melanopsin, was identified [72] and found to be exclusively expressed in the ganglion cells of RHT [20,73,74]. Melanopsin-containing RHT ganglion cells also use PACAP, another well known neurotransmitter of the RHT [20]. Melanopsin, however may not be the only circadian photoreceptor since melanopsin knock-out mice showed typical, although reduced, light responses such as entrainment and phase shifting.

Possibilities of other neuroactive substances serving as neurotransmitters in the RHT in addition to glutamate and PACAP, which are the most important candidates, have been indicated. Projections of substance P (SP)-containing ganglion cells to the ventrolateral part of the SCN have been demonstrated in lesion experiments in the rat [75]. Electrophysiological investigations also further support the role of substance P as an excitatory neuromodulator [76] responsible for the expression of both NMDA and non-NMDA receptor-mediated components of RHT transmission. Moreover, it is also reported that SP and glutamate work as agonists upstream of glutamate [77].

### Histamine

Despite substantial evidence [17,78,79] suggesting a role for histamine as a neurotransmitter in circadian entrainment [17,78,79], its role has been underplayed. With more information available and even a suggestion that histamine may be acting as a final neurotransmitter on which photic and non-photic entrainment converge [80], there has been more attention in this direction. Histamine can induce phase shifts in circadian rhythms in a manner similar to that of light pulses. Intracerebroventricular injection of histamine is also found to alter circadian function [81]. Direct effects of histamine on SCN neurons have been shown in vitro either as inhibitory or excitatory depending on experimental conditions [82,83]. It is also reported that at the level of the SCN the direct excitatory effects of histamine on neuronal firing is mediated via H_1 _receptors and the inhibitory effects via H_2 _receptors [82,83]. However, in vivo studies, it has been shown that the effects of histamine on circadian rhythms may be mediated through receptors other than histamine receptors [84,85]. The foregoing discussion supports the view that histamine may exert modifying effects on circadian rhythmicity as well as neuronal excitability. There is a clear circadian rhythm in the histaminergic activity, with high levels during the active period and low levels during the sleep period. Maintenance of circadian rhythmicity of sleep-wakefulness cycles, food intake, motility and adrenocortical hormone release seems to depend on histaminergic activity. Thus, although evidence is accumulating for a role of histamine in circadian function, it is difficult to assign it a specific role in circadian activity at this time.

### Neurotensin (NT)

Cell bodies of the rat SCN contain the neuropeptide neurotensin (NT) and two NT receptor types, namely NTS1 and NTS 2 [86-88]. In humans there is a larger population of NT neurons as compared to monkeys and other animals. Although involved in many physiological processes, the role of NT in circadian rhythm is not completely known at present. Meyer-Spasche and co-workers [89] reported that NT can phase shift the firing rate rhythm of SCN neurons. They also provided evidence that NT may play a role in regulating the circadian pacemaker through NTS1 and NTS2 receptors. NT-binding sites found in the ventral region of the SCN, which receives photic and non-photic information, is indicative of the involvement of NT in the synchronization of clock to these environmental stimuli [90]. Studies using NTS1 and NTS2 agonists, neurotransmitter receptor antagonists, as well as the exogenous application of NT, have yielded some valuable results. An increase in discharge rate of SCN neurons was observed on NT application [90]. NT-mediated effects on SCN neurons seem to result from activation of NTS1 and NTS2 receptors rather than involve glutamate or GABA receptors or modulation of the synaptic release of glutamate or GABA [90]. NPY, which is an established neurotransmitter of the geniculohypothalamic tract (GHT), was found to regulate SCN neuronal activity [91-93] and to produce long lasting suppression of firing rate of SCN neurons. When co-applied with NPY, NT was found to damp the profound inhibitory effect of NPY [90,92,93]. This is interesting since there are studies showing that NPY immunoreactive terminals overlap with NT-binding sites in the ventral part of the SCN. This was considered as evidence of an interaction of NPY and NT to regulate neural activity. From a developmental point of view, NT-expressing neurons developed earlier than the other 3 types of peptidergic neurons, NPY, VIP and AVP [94]. It remains to be seen whether NT-expressing neurons contribute significantly to the generation of circadian rhythms in early human life.

### Neuromedin S (NMS)

A recent addition to the ever increasing list of neurotransmitters of the SCN is neuromedin S (NMS), a 36 amino acid neuropeptide. It is a potent brain-gut neuropeptide whose presence in the SCN was reported by Nakahara and co-workers [95] as a neurotransmitter of the circadian oscillator system. NMS expression is found to be restricted to the core part of the SCN and has a diurnal peak under light-dark cycle [96]. Intracerebroventricular administration of neuromedin S in rats activates SCN neurons and has the capability to induce non-photic type of phase shifts in the circadian rhythm of locomotor activity. It is also possible that NMS along with VIP may have a role in maintenance of circadian rhythmicity. Recently, it has been shown that neuromedin M (NMU) is regulated in a circadian manner with peak expression in the light phase of LD cycle [97]. Further studies are required to understand the specific role of NMS in the SCN. At present, it is implicated in the regulation of circadian rhythms through autocrine and /or paracrine actions through its receptors [96].

### Gastrin releasing peptide (GRP)

Gastrin releasing peptide (GRP) has also been identified as a neurotransmitter in the SCN. Although GRP and its receptor BB_2 _are found to be synthesized by rodent SCN neurons [98-100], the role of GRP in circadian rhythm regulation is not well known. Evidence points towards a role for GRP in photic entrainment [101,102], in spite of a number of studies favouring glutamate as the main neurotransmitter [36,37,103]. McArthur and co-workers [104] studied the role of GRP in photic entrainment by using the resetting actions of GRP application on electrical activity rhythms during subjective day, early subjective night, and late subjective night in vitro in rats and hamsters. Their studies have shown phase delay on SCN neuron firing during early subjective night and phase advance during late subjective night with no response on application during subjective day. Phase shifts were blocked by a BB_2 _receptor antagonist, thereby confirming the role of GRP in the participation of photic entrainment. GRP found within calbindin-containing retinorecipient cells and also causing photic-like phase shifts on application directly to the SCN may be a possible neurotransmitter for intra-SCN communication [105].

### Acetylcholine (ACh)

Acetylcholine (ACh) has the distinction of being identified as the first neurotransmitter for the regulation of circadian rhythms. There is evidence in favour and against acetylcholine as a neurotransmitter in the literature. It was suggested that acetylcholine plays a role in the light-input pathway on the basis of some of the studies [16,106]. Electrophysiological studies indicating excitation of some neurons of the SCN by cholinergic agents [107,108] have supported the role of acetylcholine. Use of the acetylcholine receptor agonist carbachol, a non-specific agonist, to mimic the effects of light [109] also added to the supporting evidence for the role of acetylcholine in the SCN. The effect is mediated by muscarinic receptors of the M1 subtype [110]. Intraventricular administration of carbachol, which caused phase shifts in vivo, could be blocked by GluR antagonists [111]. However, acetylcholine does not appear to be directly involved as a neurotransmitter in the light-input pathway. It may act to modulate the photic information reaching the SCN.

## Geniculohypothalamic tract

The geniculohypothalamic tract (GHT) is a second afferent photic projection from the intergeniculate leaflet (IGL) to the SCN. The IGL receives input directly from the retina via a separate branch of the RHT. The projection from IGL via GHT terminates in the areas of the SCN that overlap the direct RHT-SCN input. GHT provides a secondary, indirect photic input as well as an alternate input which has an important role in entrainment mediated by non-photic stimuli such as motor activity. While lesions of the IGL block activity induced phase shifts [112], electrical stimulation produces phase shifts similar to those produced by activity [113]. It has been reported that IGL mediates photoperiodic responses as well as non-photic entrainment of circadian rhythms [114,115]. Hence, the IGL may have integration of photic and non-photic information as its function. In addition to neuropeptide Y (NPY), GHT may also have GABA and enkephalin (ENK) as neurotransmitters in rat and hamster [116,117]. ENK was found in cell bodies in the IGL as well as in fibres in the SCN of many mammalian species. High density of delta opioid receptors, which have the highest affinity for ENK, was detected in the hamster SCN [118] and an ENK agonist phase-advanced hamster wheel running activity late in subjective day [119].

### Neuropeptide Y (NPY)

RHT and GHT exhibit partial overlapping in the SCN. The SCN exhibits immunoreactivity for NPY [120]. There is evidence that IGL mediates both photoperiodic and non-photic entrainment of circadian rhythms [114]. NPY, the primary transmitter of GHT, acts directly on pacemaker neurons of the SCN in hamsters [121]. IGL neuron projections to the SCN also have GABA/NPY immunoreactivity and those projecting to contralateral IGL have GABA/ENK immunoreactivity [116,122]. There may be co-localization of NPY and GABA in the GHT projections. Since many features of the response to light by the circadian system remain unaffected by IGL lesion in animals, it is suggested that this pathway may not be critical for the photic regulation.

Further evidence is available to emphasize the importance of GHT to cause non-photic phase shifts during the day but not during the night, such as the phase-shifts evoked by activity induced by novel stimuli [123,124]. The phase shifts are abolished by IGL lesions. Both in vitro and in vivo NPY administration produced a similar pattern of phase shifts during the day, which was blocked by bicuculline [125]. NPY has also been found to act presynaptically to inhibit GABA-mediated synaptic transmission through inhibition of calcium currents [126].

### Serotonin (5HT)

A dense, robust serotonergic projection from midbrain raphe nuclei terminating predominantly in the retinorecipient region of the SCN has been reported [127]. A reciprocal projection from the SCN to raphe nuclei is also seen [128]. Both in vitro and in vivo, 5HT receptor agonists are found to cause phase shifts of the SCN when administered at times in the circadian cycle during which light does not cause phase shifts [129,130]. Raphe nuclei lesions reduce the amplitudes or "clarity " of rat's circadian activity rhythm [131] with detectable persistent rhythmicity. Serotonergic projection to the SCN terminate to a great extent on vasoactive intestinal peptide(VIP)-containing neurons in the ventrolateral part of the SCN. There is a close relation between retinal afferents and VIP-containing neurons of the SCN in this area [132].

The major function of the serotonergic projection most probably is the modulation of the pacemaker responses to light. Raphe nuclei receive retinal afferents [133] and hence the raphe-retina projection may be viewed as another indirect photic input to the biological clock. In in vitro studies, serotonin advances the phase of the circadian pacemaker during the day and delays it at night, an action similar to that of GABA [134]. Serotonin is also found to regulate SCN neurons by both pre- and post synaptic inhibitory mechanisms [135]. 5HT and 5HT agonists are also found to inhibit optic nerve-induced field potentials in the SCN brain slice preparation, and light-induced Fos expression and phase shifts of the circadian rhythm of wheel-running activity [136]. It is also reported that 5HT antagonists enhance light-induced increases in the firing rates of SCN neurons [137] and light induced phase shifts [138]. Many of the above studies point towards the hypothesis that the serotonergic innervation of the SCN serves to modulate light-induced glutaminergic input. Considering these facts, there exists a possibility of the involvement of 5HT in tonic inhibition of the light-input pathway to the SCN. There is a suggestion that this serotonergic projection from raphe to the SCN may be the anatomical substrate for affective disorders to alter human circadian system/rhythms. This belief is further strengthened by the observation that dysfunction of serotonergic pathways play a role in affective disorders and that these disorders are frequently treated with agents that alter serotonergic neurotransmission. Further studies about this pathway are likely to give more information about the link between disruptions of circadian function and affective disorders.

### GABA

It is now widely accepted that gamma amino butyric acid (GABA) is an important neurotransmitter of the SCN for regulating SCN function. Most SCN neurons express the neurotransmitter GABA and are thus GABAergic [12]. GABA receptors and receptor subunits have been described by Castel and Morris [139], Naun and co-workers [140], van den Pol [141], Gao and co-workers [142], and O'Hara and co-workers [143]. In most of the brain regions, GABA primarily acts through interaction with GABA_A _and GABA_B _receptors and produces neuronal inhibition through membrane hyperpolarization and increased membrane conductance. Glutamic acid decarboxylase (GAD) is the enzyme required for synthesizing GABA and is found in nearly all neurons of the SCN [116]. Additional support for an inhibitory role of GABA in the rat SCN has come from the studies of Gribkoff and co-workers [144,145]. However, recent investigations have shown that GABA has dual effects on the SCN neurons, excitatory during day and inhibitory at night [146], and this has been attributed to changes in [Cl^- ^]_I _during the circadian cycle. This dual inhibitory [147] and excitatory [148] action of GABA has been thought of as the probable reason for the synchronization of spiking in the SCN neurons. Under some circumstances, such as in early development, GABA can also be depolarizing and potentially excitatory [126,149-151]. The excitatory effect of GABA on SCN neurons in the night seems to be complex. While the action of GABA in the day on SCN neurons is uniformly inhibitory, the effects of GABA during the night are heterogeneous due to both depolarizing and hyperpolarizing effects. The GABA-mediated depolarizing effect seen at night is restricted to a subset of SCN neurons. Differential day-night modulation of GABAergic neurotransmission seen in the SCN may provide a time-dependent gating mechanism to counteract propagation of excitatory signals throughout the biological clock during the day and to promote it at night [152]. GABA does not seem to be synthesized in the SCN in a circadian fashion, but in a diurnal pattern as per GAD m RNA basis [153,154]. A circadian rhythm in GABA transmission in the dorsal part of the mouse SCN, with requirement of VIP for the expression of this rhythm, is reported by Itri and co-workers [155]. While considering the action of GABA in the SCN, whether inhibitory or excitatory, extrinsic GABA sources such as from IGL [116] and release of GABA from SCN terminals implicated in transmission of light information should also be looked into to visualize a clearer picture. GABA receptors and receptor subunits are expressed in the SCN [116,139,140]. Although no variation in concentration of GABA in the SCN has been reported, responsiveness of the SCN undergoes daily variation [156].

## Neurotransmitters for intra-SCN communication

Integrated output as a result of integrated activity within the SCN in spite of heterogeneity in functional and neurochemical organization explains the efficiency of the SCN, the biological clock. Such a process is likely to involve much coordinated activity, and intra-SCN communication must be strong enough to produce such an action. It is clear that photic information received must be relayed from retinorecipient cells to the oscillator cells in the nucleus. Intra-SCN signals underlying such communication are not known with certainty as yet. The importance of circadian synchrony of the SCN neurons in the normal working of the clock has been highlighted in some animal studies recently [157]. In these studies, loss of coherent daily rhythms has been shown to coincide with loss of circadian synchrony among the constituent neurons [157]. Neurotransmitters have been designated as potential SCN synchronizers and one of them, which is unique since it is expressed by most of the SCN neurons, is GABA. This and other transmitters involved in synchronization, such as VIP, GRP and prokineticin 2, have been studied quite extensively. However, the potential of the latter two, GRP and prokineticin 2 as synchronizing factors require further investigation [23]. Further, it is reported that neurotransmitters released by neurons of the ventral part of the SCN is necessary for maintaining synchrony of the whole SCN [23 ].

### Vasoactive intestinal polypeptide (VIP)

VIP, a gut polypeptide, has been identified as one of the main neurotransmitters of SCN neurons and participates in SCN function. These SCN neurons are retinorecipient and are found in the core of the SCN. They are activated by light, and exogenous application of VIP can reset the circadian clock in a manner similar to that of light application, both in vitro and in vivo[6]. It is estimated that 9%–24 % of SCN neurons express VIP [26,158]. It appears that in rats there are two types of VIP neuronal components[159], namely a medial GRP-free group and a lateral group containing GRP. Only the lateral group expresses *per1 *following a light pulse [159]. However, few VIP-containing cells rhythmically express *per1 *and *per2 *[160,161]. VIP is synthesized from prepro VIP and further cleavage of the molecule forms VIP and peptide histidine isoleucine (PHI). PHI is found in abundance in the SCN and is co-localized [162,163]. VIP and PHI are structurally related to PACAP. The receptor for VIP, VPAC_2,_also known as Vipr2, is expressed in about 60% of the SCN neurons, which respond to VIP with changes in firing rate [164,165]. VIP acting through VPAC_2 _can participate in both resetting by light and maintenance of ongoing rhythmicity in the SCN [6].

VIP along with GRP and AVP show circadian variation in the level of mRNA in constant environmental conditions [166]. Some earlier studies [167-169] had indicated that VIP and GRP do not show circadian rhythms in DD and only daily rhythms in LD. On the basis of their study, Shinohara et al [168] suggested that changes in the peptide content by light conditions might reflect changes in the synthesis and release of peptides. The release of these peptides also shows circadian variation [170]. It has been reported that treatment of SCN slices with VIP produces phase shifts similar to those induced by light pulses [171]. Nielsen and co-workers [172] showed that VIP induces *per1 *and *per2 *gene expression in rat SCN in a phase dependent manner. More recently, VIP has been shown to be necessary for the coordination of the daily rhythms in behaviour and physiology at the level of biological clock in mice [173]. Loss of internal desynchronization and its subsequent restoration were achieved by adding VIP into the mice cells. Thus, VIP signalling through its receptor serves two important functions in the SCN, namely, circadian rhythmicity in a subset of neurons and maintenance of synchrony between intrinsically rhythmic neurons. This may also mean that VIP-expressing neurons themselves are circadian pacemakers in the SCN for establishing and synchronizing rhythmic activity.

### Vasopressin (AVP)

AVP neurons occupy a large part of the SCN, mostly in the dorsomedial part of the SCN and are extensively interconnected [174,175], indicating the capacity of the SCN to produce an integrated output. It is estimated that nearly one third of the SCN neurons in rats synthesize AVP. It is one of the major neuropeptides identified in the SCN [16,176,177]. AVP is synthesized and secreted by the SCN in a circadian pattern. AVP has an important excitatory role by activating V1a receptors [177] to increase the amplitude of firing rates in the SCN during subjective day [178,179] and enhance SCN output [176,177]. Although the presence of AVP at the level of the SCN may not be critical for the expression of some of the circadian rhythms, abnormalities can be seen in some of the expressed rhythms in its absence. AVP-deficient Brattleboro rats have served as an excellent model for the demonstration of the absence of AVP and subsequent disturbances in many of the circadian rhythms. Local application of AVP into the SCN does not affect the free running circadian wheel running rhythm in hamsters[180] or entrained circadian food intake [181] and water intake [182]. A convincing supportive role for vasopressin in SCN circadian function has come from transplantation studies of DeCoursey and Buggy [183] as well as that of Lehman and co-workers [184]. Infusion of V1 receptor antagonist has been reported [185] to produce no significant effect on the wheel running activity in rats, thereby indicating no role for VP in the generation of circadian rhythms. Boer and co-workers [186] reported that vasopressin may not be a critical component in the maintenance or in the transfer of circadian activity of the biological clock for drinking activity based on their graft transplant study. In depressed patients, both synthesis and release of AVP in the SCN is reduced, which leads to an impaired functional activity of the circadian clock [187], although there was an increase in the number of AVP-immunoreactive neurons. Arima and co-workers [188] reported AVP transcription in the SCN in long-term organotypic cultures. Transcription exhibits circadian rhythmicity and is dependent on the ongoing electrical and synaptic transmission in the cultures.

It was mentioned earlier that the predominant excitatory actions of AVP within the SCN are mediated by V1 receptors, although it is not yet known with certainty whether V1a or V1b subtypes are involved in the action. Decrease in the AVP neurons and AVP content in the SCN has been reported [189-191], and this has been correlated with decreased amplitude of activity rhythms, increased rhythm fragmentation, and disruption of the normal sleep/wake cycle. However, Hochstetler and co-workers [192] did not find a correlation between differences in activity level and circadian expression and differences in the number of AVP-immunoreactive cells in the SCN. In a study by Kalamatianos and co-workers [193], it was reported that there is a decrease in the amplitude of the daily rhythm in the expression of V1a receptor mRNA along with persistently elevated level for V1b mRNA in aged male rats as compared to young adult ones. A role for AVP in the SCN not only in circadian timing but also in the circadian memory of radical events has been reported by Biemans and co-workers [194]. There have been many attempts in the past to link AVP in the SCN to specific clock function. However, the attempts have not yielded definite results so far. Reduction of AVP neurons of the SCN has been reported to eliminate or reduce the amplitude of many rhythms studied. But at the same time studies in Brattleboro rats have shown that AVP may not be necessary to maintain coherent circadian rhythmicity [195,196]. In house mice, Hochstetler and co-workers [192] reported that there is no relationship between AVP neurons in the SCN and circadian features of wheel running activity. In addition, the SCN also participates in the communication with the rest of the brain. One such output signal, primarily electrical but not exclusively, is AVP [197]. Correlation between SCN-AVP expression and circadian organization of locomotor behaviour has been shown across species including rats [198] and hamsters[199]. However, transplantation studies indicate some other diffusible factor other than AVP in the regulation of circadian rhythmicity [186].

### Melatonin

Melatonin, the hormone from the pineal gland, called the "darkness hormone " is of great importance in the functioning of the SCN. The most important target of melatonin in humans appears to be the SCN, as the SCN contains the highest density for melatonin receptors [200]. A double effect of melatonin in the SCN, namely, an immediate effect and long term effect, has encouraged its worldwide use against the ill effects of jet lag. As an immediate effect, melatonin is found to suppress neuronal SCN activity towards night time levels [201]. It also lowers VP secretion from SCN neurons as shown by experiments in rats [202]. Acceleration of sleep initiation in humans at circadian phases when the SCN would normally stimulate waking is another reported action of melatonin [203]. In terms of long term effect, melatonin can phase shift and amplify circadian rhythmicity of the SCN. Melatonin application has been found to be useful in synchronizing the endogenous circadian rhythms not only in people who suffer from jet lag, but also in blind individuals [204,205], patients with dementia [206], and shift workers [207]. Probably recognizing the importance of melatonin as a chronobiotic, many researchers have studied the applications of melatonin on human circadian rhythms. Recently, Revell and co-workers reported that administration of a combination of morning intermittent bright light and afternoon melatonin along with a gradually advancing sleep schedule can advance circadian rhythms almost an hour a day, with very little circadian misalignment [208]. This protocol might be applied before eastward jet travel or for delayed sleep phase syndrome to evoke a phase advance of the circadian clock [208]. In spite of the experimental evidence favouring a very important role for melatonin in the circadian timing system, the exact role of melatonin has not been demonstrated clearly. Melatonin and seasonal rhythms are intimately related in mammals, and this has been well documented [209,210]. Lincoln and co-workers [211] provided evidence for a temporal melatonin-controlled expression of clock genes in specific calendar cells. The retinohypothalamic -pineal (RHP) axis is comparable in animals and humans. In both animals and humans melatonin is secreted exclusively at night. The RHP is capable of detecting changes in night length to make proper adjustments for the duration of nocturnal melatonin secretion so that animals can use this melatonin message to trigger seasonal changes in behaviour [209]. With seasonal changes in night duration, there are parallel changes in the duration of melatonin secretion, and this leads to more secretion as compared to summer.

## Neurotransmitters in efferent projections

The output of the SCN by way of efferent projections serves the purpose of conveying the information to the related centres. Outputs are primarily seen to the nearby hypothalamic and thalamic nuclei from the SCN, particularly to the medial preoptic nucleus, the medial part of the paraventricular nucleus of the hypothalamus, the anterior part of the paraventricular nucleus of thalamus, the medial part of the dorsomedial nucleus of hypothalamus, and principally the subparaventricular zone [212,213]. Projections to the ventrolateral preoptic nucleus from the dorsomedial nucleus, the preoptic nucleus and the subparaventricular zone appear to serve as the anatomical basis for the control of sleep and wakefulness, as the ventrolateral preoptic nucleus is implicated in the control of sleep states [214-216]. Efferent projections seem to have mainly AVP and VIP as transmitters. These fibres that originate in the SCN can be seen for long distances within the hypothalamus and have a characteristic morphology. The functional significance of these projections remains to be fully determined, apart from the basic fact that they are necessary for the SCN to exert its overt function. The functional role has been described to some extent earlier [217].

**Figure 2:**
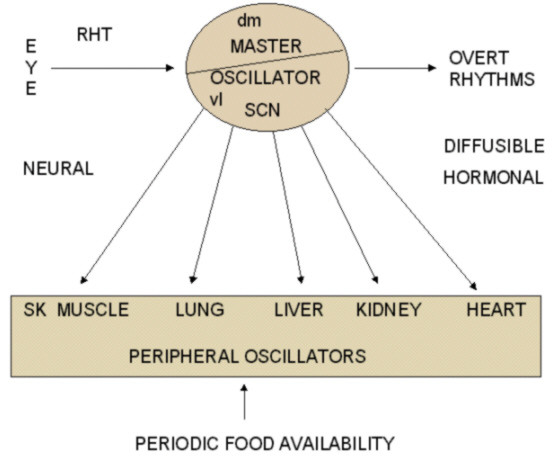
**Inter-relation and efferent outputs between central and peripheral oscillators. **Notice the neural and diffusible control of the central oscillator. Peripheral oscillators respond to signals from SCN as well as to other inputs like periodic food availability. Diffusible output may have AVP, VIP, prokineticin, and TGF-α. RHT: Retinohypothalamic tract, dm: dorsomedial SCN, vl: ventrolateral SCN.

Although the SCN is often designated as the "master" circadian pacemaker that drives most, if not all, rhythmic physiological processes, the importance of oscillators outside the SCN cannot be ignored. Indeed, there is considerable evidence for the existence of circadian pacemakers outside the SCN [218-220] (Figure 2). It is believed that the master circadian pacemaker (the SCN) has peripheral "slave" oscillators that may be individual clocks. It is necessary in such a situation to have a mechanism by which peripheral oscillators are coupled to the master oscillator thereby synchronizing the activity of an organ with the central clock. A humoral substance mediating the circadian signal may be available in the efferent output in such case [221]. One such recently identified diffusible output candidate from the SCN is transforming growth factor α (TGFα) [222]. TGFα is found extensively in the brain and is a member of the epidermal growth factor (EGF) family produced by both neurons and astrocytes [223]. In situ hybridization and immunocytochemistry techniques have demonstrated the presence of TGFα in the SCN of rats [224,225] and Syrian hamsters [222,226,227]. Van der Zee and co-workers [228] reported that the two output systems of the SCN, namely AVP and TGFα, are anatomically separate, having different daily profiles in expression.

SCN output pathways in addition to influencing the hypothalamic neighbourhood [229] can be traced to extra hypothalamic sites as far as the liver, thyroid, adrenal, and salivary glands in rats [230,231]. Both neural output pathways and diffusible non-neural pathways become important in elucidating the functional significance of SCN output in terms of the control of the SCN on other oscillators.

Though the various mechanisms used to regulate the activity of other systems of the body is unclear at present, a number of hormones with direct actions on different parts of the body are produced by the SCN. These include AVP, VIP, GRP, and SS. A review by Van Esseveldt and co-workers [9] describes not only the transmitters of RHT but also the output of the SCN. A long neural signalling pathway from the SCN regulates the pineal gland secretion of melatonin. SCN neurons also stimulate gonadotrophin releasing hormone (GnRH) synthesizing neurons of the preoptic area and thereby affect sex hormone cycles [232]. It is generally seen that outputs of the circadian system are rhythmic but not temperature compensated. In spite of the difficulties to understand the mechanisms by which the SCN regulates a wide range of physiological outputs, it has been agreed that there are two types of signals originating from the SCN. These are hormonal and neural outputs. Transplantation experiments [233] in particular have provided highly useful evidences for the suggestion that hormonal /diffusible factors produced by the SCN act as an important output signal for the circadian system [234]. Quick recovery of behavioural rhythms within 4 days of transplantation of the SCN [184], successful placement of transplants at locations distant from the SCN [184], and transplanted dissociated SCN cells capable of restoring rhythmicity [235] are all in favour of hormonal /diffusible factors as the output signal.

It is possible that different physiological systems are controlled by either neural or hormonal output from the SCN. For example, rhythmic secretion of melatonin could be under neural control while locomotor activity might be under hormonal control. Also, a specific physiological function may receive both neural and hormonal signals. As regards to the communication of information from the circadian clock to the centres controlling activity in brain areas, more than one mode is indicated [222,236].

Analysis of the chemoarchitecture of the SCN have shown that, in addition to the above neurotransmitters, the SCN also contain neurons capable of synthesizing a number of other neurochemicals, with the distribution of immunoreactive neurons differing slightly for each neurochemical. A few of these neurochemicals are described here.

### Somatostatin (SS)

SS producing neurons of the SCN are located in both the core and shell portions [237] and form a distinct peptidergic neuronal group. These cells are few in the mouse SCN, but are large in size. Part of the SS and Substance P (SP) peptides are co-localized [238]. The shell portion of the SCN, which is likely to be involved in the regulation of overt rhythms, projects within the SCN through SS fibres [239]. In an interesting study, Biemans co-workers [240] reported a significant increase in SS and SP immunoreactivity in aged Wistar rats as compared to young ones, thereby indicating that not all SCN neuropeptidergic systems decline with age. However, another study in Syrian hamsters [241] showed that there is no effect of age on SS mRNA. Aging effects of SCN neuropeptide expression, like the circadian profile of peptide expression, may be species specific as far as the SCN is concerned. Further, it has been reported [242] that SS has a role in phase shifting and is able to reset the phase of the clock [243]. Synapse of SS fibres on VIP and AVP neurons and presence of SS receptors in the SCN is suggestive of a regulatory role for SS on other peptidergic neurons. An inhibitory modulating role of SS on VIP rhythmicity is seen [244]. Increase in SS immunoreactivity could explain the observed VIP decrease with aging, and, if enhanced SS immunoreactivity reflects a release deficit, this may lead to reduction in inhibitory action [240].

### Calbindin (CalB)

Calbindin (CalB) neurons are also found throughout the core and shell subdivisions of the SCN. Silver and co-workers [245] reported the presence of CalB-containing cells in the caudal part of the SCN. CalB cells are densely packed and receive direct retinal synaptic input [246] and respond to photic stimuli. Study of Silver and co-workers [245] indicates that CalB cells are present in the input pathway for photic stimuli reaching the SCN. The CalB subregion of the SCN seems essential for the maintenance of circadian locomotor activity rhythm. This comes from the studies of lesion as well as transplantation. Animals with lesions that destroyed CalB neurons but spared other neurons of the SCN lost rhythmicity of locomotor activity, and transplants of SCN tissue containing CalB cells restored rhythmicity. SCN transplants lacking CalB cells failed to restore rhythmicity [247]. CalB cells are found to co-localize with other peptides such as GRP, SP, and VIP. Interconnections of CalB cells with AVP, GRP, CCK, NPY and VIP have been reported [246], and the pattern of interconnection of CalB cells appear specialized in the sense that some are bilateral, while others are not [248]. The strength of linkage between interconnections also varies. Intra-SCN connections are influenced to a great extent by the projections of CalB cells. The connections are direct in some cases and indirect in others. However, indirect communication as is the case with CalB to AVP is equally effective. In addition to intra SCN communication, CalB cells may also project to extra SCN regions [248]. CalB cells appear to be non rhythmic. They, however, receive photic input from the RHT and GHT, and rapid transmission of light information to CalB neurons may facilitate circadian output [226]. Light exposure always increases firing rates of SCN neurons. However, cells in the core of the SCN oscillate in their responsiveness to photic input [5]. Light also induces clock gene expression in the SCN but only during the night [8,249]. This gene expression pattern seems to be mirrored by changes in subcellular localization of CalB, the cellular nucleus being devoid of CalB during the night [249]. Behavioural and molecular responses to nocturnal light pulses also disappeared on decreasing CalB levels [249]. Thus, CalB cells in the SCN function as gates to relay photic signals when open and to block the signals when closed, which suggests a central role for CalB neurons in gating photic input. Further investigation seems essential to elucidate the relation between functional connectivity and a single coherent output from the SCN.

### Calretinin (CALR)

Calretinin (CALR) is another variety of neurons found in the SCN (in the core ventral part) that appears to be co-localized with VIP neurons. CALR cells are small to medium sized. Optic tracts also show heavy immunoreactivity for CALR. Like calbindin (CalB), CALR is a calcium binding protein. Apparently, there is a developmental reduction in Calbindin-D28k expression parallel to RHT formation and a developmental increase in calretinin expression which is independent of RHT connections to SCN neurons [250].

### Galanin (Gal)

Galanin is yet another peptide associated with the SCN. It is a 29/30 amino acid neuropeptide seen in many parts of the nervous system [251], including the SCN [9,252,253]. In fact, neurons containing both galanin and AVP have been reported in human SCN [253]. In rat SCN, galanin receptor subtype R2 (Gal-R2) expression has been identified [254].

### Angiotensin II (ANG II)

Angiotensin II (ANG II) is an octapeptide found in the SCN [15,255] and is likely to be involved in circadian function. The presence of ANG II in the SCN has been demonstrated both at light microscopic [15] and electron microscopic level [256] in normotensive rats. Of late there has been much interest in the physiological, pharmacological, and immunohistological studies of angiotensins of the SCN. At present the available information on the role of angiotensin II at the level of the SCN is much less than that on the role of known transmitters of the SCN. It has been proposed that SCN-derived angiotensin II acts as a neuromodulator as well as neurotransmitter with the effects being mediated through angiotensin1(AT_1_) receptors located on the endothelial plasma membrane of SCN parenchyma [257].

### Met-Enkephalin (mENK)

These neurons are located primarily dorsomedially in the shell of the SCN and overlap with the distribution of AVP neurons. mENK cells are large to medium sized. Enkephalin has been found in the neurons of the IGL [116,117,122,258] projecting to the ventrolateral aspect of the SCN. The role of enkephalin as a putative neurotransmitter has been demonstrated in hamster SCN. Injection of retrograde tracer fluoro-gold into the SCN showed the existence of a population of labelled neurons in the intergeniculate leaflet which are immunoreactive for enkephalin [117]. Among the three main classes of opioid receptors, little δ or μ opioid receptor expression has been identified through autoradiographic techniques in hamster SCN in vitro [259,260]. No direct effect on basal or N-methyl-D-aspartate (NMDA)-evoked firing rates of SCN neurons in hamster has been observed on short -term application of opioid receptor agonists [261]. However, the observation of SCN neurons exhibiting withdrawal responses after the influence of enkephalins had been removed indicates that endogenous opioids may play a role in modulation of SCN function. On the other hand, naloxone injection into the SCN is reported to produce disruption of the circadian pattern of food [262] and water [263] intake.

### Prokineticin 2 (PK2)

Prokineticin 2 (PK2) has been identified as an output molecule from the SCN circadian clock [236]. PK2 is a cystein-rich secreted protein. It is reported to be involved in the transmission of behavioural circadian rhythm as well as in local function within the SCN to synchronize the out put [236]. PK2, named for its ability to stimulate intestinal smooth muscle contractility, is proposed to have a major role in inhibiting locomotor activity during the day in nocturnal species [236]. Receptors for PK2, PKR2, are found abundantly in the target nuclei of the SCN output pathway, indicating again that it is an output molecule. A recent study [264] demonstrated that the molecular rhythm of PK2 in the SCN is regulated by both circadian clock and light, with the clock having a predominant role and light having a modulatory role. PK2 expression induced by light independent of the circadian oscillator may also indicate participation of PK2 in the photic entrainment of circadian rhythms. Evidence suggesting diurnality in behavioural patterns based on alterations in PK2 receptors in regions receiving SCN efferents does not seems to provide a complete answer at least in rodents[265]. The action may be downstream of the SCN or may be in parallel to the PK2/PKR2 receptor systems[265].

## Relationship between the biological clock of humans and those of other mammals

The majority of studies of SCN function has been conducted on laboratory animals, particularly the rat. Similarities in the anatomical and physiological data available strongly suggest that the SCN of humans and other mammals are functionally similar. Two unique case reports [266,267] suggested that lesions of the SCN lead to disruption of circadian rhythmicity in humans. The location of the SCN, in the anterior hypothalamus, bilaterally next to third ventricle and above the optic chiasm, and the afferent and efferent projections of the SCN are similar in humans and other mammals. In the case of neurotransmitters involved in SCN function, also similar number and well defined subdivisions can be seen. Human SCN additionally contain neurotensin as a neurotransmitter. In general, human SCN has well defined subdivisions with chemically defined neuronal groups comparable to those described in experimental animals [18]. More details can be seen in the review article by Scheer and co-workers [268]. A recent review by Bell-Pedersen and co-workers [269] summarizes the similarities and differences in different organisms and discusses the organization of the circadian system as a composite of multiple oscillators. The endogenous nature of the clock, its entrainment by time cues, its phase response curves for light and melatonin, and its electrophysiological and metabolic activity are other features that are common to humans and other mammals.

Studies on animal models have helped the understanding of many disease conditions seen in humans. The lack of proper functioning of the biological clock seen in the elderly, the ill effects of jet lag, and clinical intolerance in shift workers are all topics of current research in experimental studies. Animal experimentation with new rodent models for circadian study involving transgenic and knockout mice and rats have contributed to our understanding of the above as well as many other conditions. Fu and collaborators [270] showed that the life span of their *period2 *knockout mice was less than that of wild type sibling mice. They also reported that these mice develop spontaneous lymphomas of high frequency, which suggests that the *period2 *gene plays an important role in tumor suppression. Studies on liver regeneration after partial hepatectomy in mice showed that regeneration occurs only at certain times of the circadian day [271]. A clear mechanistic link between the clock and tolerance to anti-cancer drugs has been reported [272].

### Genes and their linkage with neurotransmitters in the working of the clock

Presence of robust, overt circadian rhythm expression by the SCN depends on a stable pacemaker with period length of approximately 24 hours. This in turn is made possible by the integration of the thousands of individual cellular clocks found in the SCN. Using real-time analysis of gene expression, Yamaguchi and co-workers [30] have shown synchronized rhythms of clock gene transcription across hundreds of neurons within the mammalian SCN. Whether it is the neurotransmitters in the afferent input to the SCN, or of the SCN itself or efferent projection from the SCN, neurotransmitters and their linkage with genes are important, and this has been the basis for the information now available on the molecular basis of the biological clock and its working.

In mammals, the molecular mechanism for generation of circadian rhythms is dependent on the concerted co-expression of specific clock genes [273]. The genes in mammals are the period (*per1*, *per2*, and *per3*), cryptochrome (*cry1 *and *cry2*), clock (*clk*), and brain-muscle-Arnt-like-protein 1 (*bmal1*) genes. It is now known that light entrainment of the clock involves the induction of c-Fos [274] and clock genes *per1 *and *per2 *[275-277]. These genes are rapidly induced in the SCN by light stimulation at those time points at which light phase shifts the clock. Possible genes for glutamate and PACAP thus appear to be the clock genes *per1 *and *per2*, which are induced in the SCN by light, glutamate and PACAP at night. Further, Nielsen and co-workers [172] also have shown that VIP induces *per1 *and *per2 *expression in a phase-dependent manner, thereby suggesting that VIP is important for the light-induced phase shift at night.

### Neurotransmitters and disease conditions

Researchers have started to identify the role of the SCN in certain disease conditions. SCN dysfunction, particularly in terms of neurotransmitter content, has been associated with several chronic diseases such as hypertension, diabetes, and depression [278,279].The anatomical picture with respect to staining in the SCN is changed in spontaneous hypertensive rats (SHR), and transplantation of hypothalamic tissue containing the SCN from SHR to normotensive rats induces hypertension [280]. Decrease in staining for many SCN neurotransmitters along with an enhanced activity of paraventricular nucleus (PVN) CRH neurons have been observed in hypertensive patients [281]. This suggests that, in humans, the SCN may have an inhibitory role in the CRH neurons of the PVN. These observations strongly suggest that a changed SCN may precede the development of hypertension. There is also evidence that circadian disturbances may be detected prior to the development of diabetes or hypertension [282,283]. Further evidence that the functionality of the biological clock may be affected in humans by diseases such as depression and hypertension has been provided by post-mortem analysis of the SCN in human physiological disorders by Zhou and co-workers [187] and Goncharuk and co-workers [280]. One of the possible explanations for the development of hypertension may be that a less active SCN may prepare an individual less effectively for the new period of activity, and that repetition of this strain over the years may result in hypertension. One can find support for this theory in the observation that cardiovascular accidents precipitate during morning hours when the onset of activity occurs.

## Conclusion

Neurotransmitters of the circadian clock have been investigated more and more with the unfolding of the understanding of the clock, particularly at the molecular level. From a time when hardly anything was known about neurotransmitter involvement in the working of the clock, we have come to a stage of controlled manipulation on the basis of the properties and nature of neurotransmitters. The ill effects of jet lag, clinical intolerance to shift work, disruptions of the working of the clock with old age and their correction to some extent with the help of chemicals, especially those like melatonin, will hopefully be treated in the near future by interventions developed with knowledge of SCN neurotransmitters. Chronotherapy has become an advantageous therapeutic option for many disease conditions. Chronomodulation methods for chemotherapy, radiotherapy and even immunotherapy have been highlighted by many researchers and physicians in the recent past for treatment of patients with cancer. With the advances in molecular chronobiology and clarification of its mechanisms, chronotherapy is likely to become increasingly accepted as a effective means for treating not only cancer but also many other disease conditions. Thus, knowledge of the workings of the clock and its neurotransmitters appears to be essential not only for disease prevention but for therapeutic practice as well.

## Competing interests

The author(s) declare that they have no competing interests.

## Authors' contributions

**VR **and **RR **contributed equally to this work (literature search, systematization, and writing).
